# Semi-Supervised Class-Incremental Sucker-Rod Pumping Well Operating Condition Recognition Based on Multi-Source Data Distillation

**DOI:** 10.3390/s25082372

**Published:** 2025-04-09

**Authors:** Weiwei Zhao, Bin Zhou, Yanjiang Wang, Weifeng Liu

**Affiliations:** 1 School of Computer Science and Technology, Shandong University of Technology, Zibo 255000, China; 2 College of Control Science and Engineering, China University of Petroleum (East China), Qingdao 266000, China

**Keywords:** sucker-rod pumping well operating condition recognition, multi-source data fusion, attention mechanism, distillation learning, label propagation, graph neural network

## Abstract

The complex and variable operating conditions of sucker-rod pumping wells pose a significant challenge for the timely and accurate identification of oil well operating conditions. Effective deep learning based on measured multi-source data obtained from the sucker-rod pumping well production site offers a promising solution to the challenge. However, existing deep learning-based operating condition recognition methods are constrained by several factors: the limitations of traditional operating condition recognition methods based on single-source and multi-source data, the need for large amounts of labeled data for training, and the high robustness requirement for recognizing complex and variable data. Therefore, we propose a semi-supervised class-incremental sucker-rod pumping well operating condition recognition method based on measured multi-source data distillation. Firstly, we select measured ground dynamometer cards and measured electrical power cards as information sources, and construct the graph neural network teacher models for data sources, and dynamically fuse the prediction probability of each teacher model through the Squeeze-and-Excitation attention mechanism. Then, we introduce a multi-source data distillation loss. It uses Kullback-Leibler (KL) divergence to measure the difference between the output logic of the teacher and student models. This helps reduce the forgetting of old operating condition category knowledge during class-incremental learning. Finally, we employ a multi-source semi-supervised graph classification method based on enhanced label propagation, which improves the label propagation method through a logistic regression classifier. This method can deeply explore the potential relationship between labeled and unlabeled samples, so as to further enhance the classification performance. Extensive experimental results show that the proposed method achieves superior recognition performance and enhanced engineering practicality in real-world class-incremental oil extraction production scenarios with complex and variable operating conditions.

## 1. Introduction

In the process of oil extraction and production, the sucker-rod pumping well operating condition recognition is crucial for efficient oilfield production [1,2]. As a nonlinear machine-rod-fluid coupled system, the operating condition is dynamically influenced by multiple factors, such as geological conditions, equipment parameters, and other interacting factors. This complex nonlinear interaction results in exhibiting multi-source, high-dimensional, and dynamically variable characteristics. With ongoing oilfield development, the sucker-rod pumping well operating conditions are becoming increasingly complex and variable, requiring recognition models to adapt to new conditions while maintaining accuracy for existing ones. However, most existing operating condition recognition methods are based on fixed-category assumptions [3], exhibiting significant limitations when addressing class-incremental problems. Specifically, the limitations include: (1) poor robustness: recognition performance degrades significantly for new condition categories. (2) the lack of effective knowledge retention mechanisms makes it difficult to ensure the recognition accuracy of existing condition categories. The integration of deep learning and class-incremental learning can effectively address the limitations of existing methods in dealing with class-incremental problems [4,5].

In the context of big-data-driven oil production, sucker-rod pumping well systems acquire massive multi-source measured data, such as dynamometer cards and electrical parameters. Additionally, a substantial volume of unlabeled operating condition data has also been obtained, which provides a rich foundation for operating condition recognition. Current deep learning-based methods for sucker-rod pumping well operating condition recognition primarily fall into two categories: methods based on single-source data and multi-source data. Methods based on single-source data typically focus on extracting features from individual data types (e.g., dynamometer cards or electrical parameters) [6,7] and employ deep learning models for classification. Methods based on multi-source data integrate diverse data streams, including dynamometer cards, electrical parameters, and production parameters (e.g., production rate, pumping metrics, wellbore data). While these methods have achieved some success, several challenges remain: (1) Most methods require extensive labeled training data and fixed category assumptions, limiting their adaptability to new conditions. (2) Single-source limitations: Approaches relying on single data sources are prone to false alarms in complex scenarios. (3) Multi-source methods face performance and robustness issues due to limitations in traditional fusion techniques and statistical uncertainties in production data.

Graph neural networks (GNNs) demonstrate remarkable capabilities in structural modeling and relational reasoning by transforming high-dimensional complex data into graph-structured representations. This unique modeling method endows GNNs with significant advantages in handling multi-source, high-dimensional, and dynamically changing graph data. In the field of semi-supervised deep learning, graph-based label propagation methods [8] have achieved notable progress in recent years. These methods primarily operate by propagating label information through graph structures to simulate complex relationships between data points, enabling models to learn from limited labeled data and generalize to unlabeled instances. Studies have demonstrated that integrating label propagation with distillation learning can effectively address class-incremental learning challenges [9]. However, label propagation-based graph learning methods exhibit certain limitations when processing multi-source data. Most existing methods are primarily designed for single-source data scenarios, failing to fully leverage the rich information contained in multi-source datasets. As different data sources may contain complementary information, current methods lack effective fusion strategies to integrate features from heterogeneous information sources, which consequently constrains further improvements in model performance.

To address the challenges, we propose a semi-supervised class-incremental sucker-rod pumping well operating condition recognition method based on measured multi-source data distillation. Firstly, we select measured ground dynamometer cards and measured electrical power cards as information sources, and construct the graph neural network teacher model for each data source, and dynamically fuse the predicted probabilities of each teacher model using the squeeze-and-excitation attention mechanism. This integration strategy not only enhances the model’s adaptability to multi-source data but also effectively captures the inter-source complementary information. Subsequently, to address the catastrophic forgetting problem during class-incremental learning, we introduce a multi-source data distillation loss, which employs the Kullback-Leibler divergence to measure the differences between the output logic of the teacher and student models. This helps the student model retain the knowledge of old categories while learning new ones, thereby mitigating the forgetting of knowledge about old categories in the class-incremental learning process. Finally, we employ a multi-source semi-supervised graph classification method based on enhanced label propagation. By improving the method with a logistic regression classifier, it deeply explores the potential relationships between labeled and unlabeled samples, further enhancing the effectiveness of operating condition recognition.

The main contributions of this paper are as follows:

- This paper proposes a measured multi-source semi-supervised class-incremental framework, which effectively enhances the model’s recognition performance and robustness under complex and dynamic operating conditions by leveraging the complementary nature of multi-source data, the anti-forgetting characteristics of class-incremental learning, the efficiency of semi-supervised learning, and the structural modeling capabilities of graph neural networks.
- For the class-incremental sucker-rod pumping well operating condition recognition task, we introduce a multi-source data distillation method. It uses Kullback-Leibler divergence to measure the differences between the output logic of the multi-source teacher and student models, thereby alleviating the forgetting of old category knowledge during the class-incremental learning process and overcoming the limitations of traditional fixed-category classification methods.
- By dynamically integrating the predicted probabilities of each teacher model through the Squeeze-and-Excitation attention mechanism, the model’s adaptability to multi-source data is enhanced, effectively capturing the complementary information between multi-source datasets.
- Through extensive experimental evaluation, the proposed method demonstrates superior performance on class-incremental sucker-rod pumping well operating condition recognition datasets, further improving the accuracy and robustness of sucker-rod pumping well operating condition recognition.

The remainder of this paper is organized as follows: Section 2 provides a review of related works, including single-source and multi-source information-based sucker-rod pumping well operating condition recognition methods. Section 3 presents the proposed semi-supervised class-incremental recognition method based on multi-source data distillation. Section 4 describes the experimental setup and results, demonstrating the superior performance of the proposed method. Finally, Section 5 concludes the paper and highlights future research directions.

## 2. Related Works

### 2.1. Single-Source Information-Based Sucker-Rod Pumping Well Operating Condition Recognition Methods

In the field of sucker-rod pumping well operating condition recognition, dynamometer cards and electric power parameters have been widely applied in the research of operating condition recognition technology. Dynamometer cards can intuitively reflect the variation relationship between the load and displacement of the polished rod during the operation cycle of the sucker-rod pumping unit, and electric power parameters are widely adopted due to their low collection cost and high reliability. Single-source information-based sucker-rod pumping well operating condition recognition methods extract features from dynamometer cards and electric power parameters and combine artificial intelligence algorithms to achieve the recognition of sucker-rod pumping well operating conditions. Zhang et al. [10] utilized the fast discrete curvelet transform to extract features and established a condition recognition model based on the sparse multi-graph regularized extreme learning machine (ELM). Zheng et al. [11] employed the barycentric decomposition algorithm to obtain features from dynamometer card data, followed by the use of hidden markov models combined with the clonal selection algorithm for identification. Li et al. [12] used the moment feature method and the “four-point method” to extract feature parameters from dynamometer cards and then conducted working condition recognition through multi-class support vector machines combined with particle swarm optimization algorithms. Chen et al. [13] derived a transformation model for electric power and established a feature atlas of electric power for fault diagnosis. Lv et al. [14] proposed an evolutionary support vector machine method based on incremental algorithms and simulated dynamometer cards. Researchers have developed a new model to describe the working process of the sucker-rod pumping system under various fault conditions, which is crucial for generating accurate training data. Additionally, they have proposed static apparent stiffness features and their corresponding extraction algorithms to fully retain and analyze fault information present in the dynamometer cards, thereby enhancing the diagnostic capabilities of the model. By employing incremental SVM technology, the diagnostic model can adapt to the addition of new data, achieving gradual updates rather than complete retraining, thereby enhancing the model’s adaptability and generalization to new operating conditions. However, its ability to only extract shallow features hinders further performance improvement.

With the advancement of deep learning technology, researchers have begun to utilize deep learning models to autonomously learn the deep feature representations in order to enhance the performance of working condition recognition. He et al. [15] proposed a recognition method combining the 4-segment time-frequency signature matrix with deep learning, which has demonstrated superior performance. Ye et al. [16] proposed a convolutional neural network model based on AlexNet for the automatic analysis of the dynamometer cards of sucker-rod pumps. This model simplifies the recognition process by incorporating various downhole conditions into a unified framework for high-precision diagnosis, facilitating real-time adjustments to the pumping unit parameters. Wei et al. [17] developed a deep and broad learning system based on electric power data for fault diagnosis. The system employs convolutional neural networks to extract deep features from electric power signals and integrates a broad learning system as a classifier for fault diagnosis. These approaches overcome the limitations of manual analysis and traditional machine learning in feature extraction, leveraging the strengths of deep learning to automatically construct deep networks that capture deep feature representations from dynamometer cards or electric parameter data. However, these methods require a large amount of labeled condition data for model training. He et al. [18] adjusted the parameters of the convolutional shrinkage neural network through meta-learning to address the decline in recognition performance and generalization capabilities with a limited number of labeled training samples. This method uses a few-shot learning approach to alleviate the dependence on a large number of labeled data. However, the model parameters remain static and cannot adapt to the increasing categories of sucker-rod pumping well conditions.

### 2.2. Multi-Source Information-Based Sucker-Rod Pumping Well Operating Condition Recognition Methods

Multi-source information-based sucker-rod pumping well operating condition recognition methods integrate data from various sources, such as dynamometer cards, electric power parameters, and oil well production statistics, effectively avoiding the inherent limitations of identification when using single-source data. These methods can provide a more comprehensive set of operating condition information and more reliable diagnostic outcomes. Li et al. [19] proposed a sucker-rod pumping well operating condition recognition method based on the fusion of multiple features, which combines the graphic features and Fourier descriptors of the dynamometer cards. By enhancing the robustness of features through an interactive fusion module, the accuracy of fault diagnosis is significantly improved. Abdurakipov et al. [2] integrated dynamometer cards, suction pressure, and temperature production data to establish a working condition recognition model using a transformer network, further enriching the application of multi-source information fusion techniques in sucker-rod pumping well operating condition recognition. Liu et al. [20] proposed a real-time diagnostic method based on the combined analysis of multiple data for comprehensively assessing the reservoir-wellbore-surface conditions of sucker-rod pump wells. This method establishes a comprehensive diagnostic model, effectively improving the diagnostic effect and expanding the scope of diagnosis.

Zhang et al. [21] adopted feature data such as dynamometer cards, power, dynamic fluid level, liquid-producing capacity. Zhang et al. [10] introduced “power versus position plots” to enhance diagnostic accuracy and constructed a comprehensive diagnostic model that achieved a full diagnosis of the wellbore and surface conditions. Currently, most deep learning-based multi-source condition recognition technologies rely heavily on a large amount of labeled data, and multi-source fusion techniques are generally traditional. Zhou et al. [22] presented a semi-supervised condition recognition technology that integrates four types of measured data: actual dynamometer cards, electrical power signals, wellhead temperature, and wellhead pressure. This technology demonstrates good model performance and strong engineering practicality. However, existing methods face challenges in adapting to scenarios where new class samples are gradually increasing, limiting the model’s applicability and scalability in class-increment scenarios. Moreover, existing methods require training samples with fixed categories, leading to insufficient adaptability of the model when faced with new operating conditions. Therefore, further research is needed to address class-incremental learning problems, enhancing the model’s robustness and scalability in scenarios with dynamically changing operating conditions.

## 3. Method

### 3.1. Problem Definition

This paper proposes a semi-supervised class-incremental sucker-rod pumping well operating condition recognition tasks based on multi-source data and graph neural networks. It is assumed that the multi-source dataset *D* comprises *T* class-incremental learning tasks, $D={\left\{D_{t}\right\}}_{t=1}^{T}$, and each task $D_{t}$ includes data from *V* different sources, denoted as $D_{t}=\left\{D_{t}^{v}|v=1,\cdots ,V\right\}$. For each task, the data from each source $D_{t}^{v}$ is divided into two parts: labeled dataset $L_{t}^{v}={\left\{{\left(x_{t}^{v}\right)}_{i},{\left(y_{t}\right)}_{i}\right\}}_{i=1}^{k}$ and unlabeled dataset $U_{t}^{v}={\left\{{\left(x_{t}^{v}\right)}_{j}\right\}}_{j=k+1}^{k+m}$. The labeled dataset $L_{t}^{v}$ consists of *k* labeled samples, where ${\left(x_{t}^{v}\right)}_{i}$ represents the *i*-th sample data from the *v*-th data source in task *t*, and ${\left(y_{t}\right)}_{i}$ is the corresponding label for the *i*-th sample data in task *t*, with *k* being the total number of labeled data. The unlabeled dataset $U_{t}^{v}$, ${\left(x_{t}^{v}\right)}_{j}$ signifies the *j*-th unlabeled sample data from the *v*-th data source in task *t*, and *m* represents the total number of unlabeled data. $c_{t}$ indicates the number of classes contained in the data from the *v*-th data source in task *t*. Appendix A show the key symbols and variables.

### 3.2. Proposed Method

**Figure 1:**
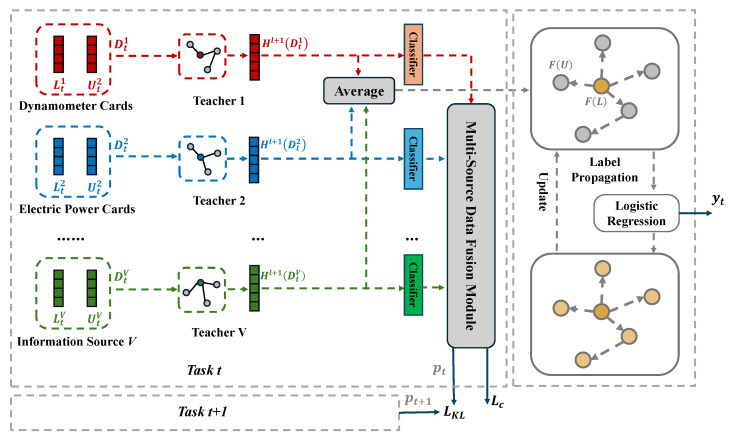
Model Architecture Diagram.

The paper proposes a semi-supervised class-incremental operating condition recognition method based on multi-source data distillation. Figure 1 illustrates the overall structure of the model, which concludes following three key steps: (1) Multi-source teacher model fusion. We construct a graph neural network teacher model for each information source, ensuring that these teacher models are inherently diverse. Each teacher model acts as a small branch integrated into the main graph neural network model, sharing most of the feature extraction layers to reduce time and memory consumption. Then, we dynamically learn the optimal weights of the predicted probabilities from each teacher model using the Squeeze-and-Excitation attention mechanism, effectively integrating the multi-source teacher models. (2) Multi-source Data Distillation Loss. With the assistance of the integrated multi-source teacher models, the student model learns the deep feature representations of the multi-source teacher models through distillation loss. The distillation loss is based on the Kullback-Leibler divergence, which measures the differences between the output logics of the teacher and student models. In this way, the student model can mitigate the forgetting of old task knowledge while continuously learning class-incremental tasks. (3) Multi-source Semi-supervised Enhanced Label Propagation Method. The method improves the label propagation algorithm with a logistic regression classifier and introduces multi-source fused features, further exploring the potential relationships between a small number of labeled samples and a large number of unlabeled samples, thereby enhancing classification performance. By using multi-source fused features as input for the enhanced label propagation method, it effectively reduces the limitations faced when constructing graph structural data relying on single-source data, such as histogram of oriented gradients (HOG) features [23], obtaining richer and more accurate node representations, and thus constructing graph structural data that more closely matches the characteristics of the actual data distribution, enhancing the model’s performance in the operating condition recognition task.

### 3.3. Multi-Source Teacher Model Fusion

Firstly, for the original binary images from each information source, we use the HOG to extract the local texture feature information of the image data. Then, we use the nearest neighbor method to construct a graph structure for each data source. Specifically, we calculated the distance between it and all other samples for each sample in a single data source, and the samples with the least distances were selected as their nearest neighbors to build the initial adjacency matrix for the graph convolutional network (GCN). The computation for the GCN of the *i*-th task is shown in Equation (1):

(1) $$G_{t}^{v}=\left(A_{t}^{v},D_{t}^{v}\right)$$

where $A_{t}^{v}\in R^{K\times K}$ is the adjacency matrix constructed for the *v*-th data source in task *t*. $D_{t}^{v}$ is the data for the *v*-th data source in task *t*, including both labeled and unlabeled data from the *v*-th data source.

The node features are then extracted using the convolutional layer of the GCN, as shown in Equation (2):

(2) $$H^{l+1}\left(D_{t}^{v}\right)=relu\left({\tilde{D}}_{t}^{-\frac{1}{2}}{\tilde{A}}_{t}{\tilde{D}}_{t}^{-\frac{1}{2}}H^{l}\left(D_{t}^{v}\right)W^{l}\right)$$

where ${\tilde{A}}_{t}=A_{t}+I_{K}$, $I_{K}$ is the identity matrix. ${\tilde{D}}_{t}$ is a diagonal matrix with diagonal elements, ${\left({\tilde{D}}_{t}\right)}_{ii}=\sum_{k=1}^{K}{\left({\tilde{A}}_{t}\right)}_{ik}$. The relu(·) function is applied for activation, *W* is the learnable parameter matrix, and *l* is the number of layers in the GCN. $H^{l}\left(D_{t}^{v}\right)$ represents the features output by the *l*-th layer of the GCN. The prediction probability $p_{t}^{v}$ by the teacher model of the *v*-th data source is calculated as shown in Equation (3):

(3) $$p_{t}^{v}=FC\left(H^{l+1}\left(D_{t}^{v}\right)\right)$$

where FC denotes the fully connected layer, which is used to classify the features output by the GCN.

**Figure 2:**
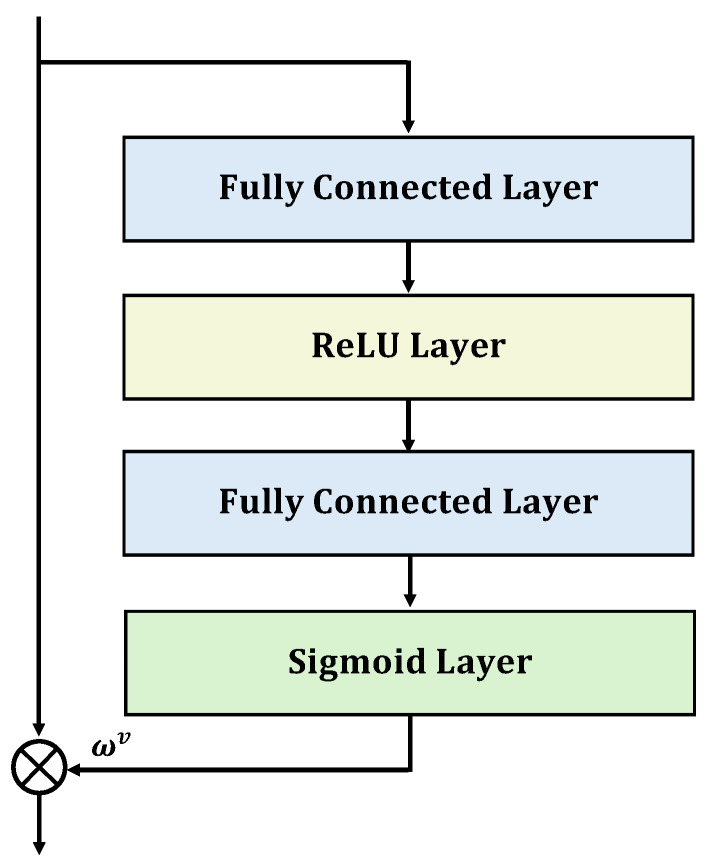
Squeeze-and-Excitation Attention Mechanism Structure Diagram.

Furthermore, we employ an attention mechanism to dynamically learn the weights of the prediction probabilities from various data source teacher models. By differentially weighting the prediction outcomes of teacher models across different data sources, we achieve an effective integration of multi-source teacher models. Specifically, this paper adapts the Squeeze-and-Excitation attention mechanism to develop a module for the fusion of multi-source feature representations. This module consists of a sequence: Fully Connected Layer, ReLU activation layer, another Fully Connected Layer, and Sigmoid activation layer, as depicted in Figure 2. The prediction probability output by the teacher model for the *v*-th data source branch is represented as $p_{t}^{v}$, with its corresponding attention score denoted by $\omega^{v}$, as defined in Equation (4):

(4) $$\omega^{v}=\mathrm{sigmoid}\left(W_{2}\left[\mathrm{relu}\left(W_{1}\left[p_{t}^{v}\right]\right)\right]\right)$$

where $W_{1}$ and $W_{2}$ are the weight matrices for the two fully connected layers, and the sigmoid(·) is the sigmoid activation function.

Next, we apply the calculated attention weights to the prediction outcomes from the multi-source teacher models to produce the integrated prediction results $p_{t}$, as detailed in Equation (5):

(5) $$p_{t}=\sum_{v=1}^{V}\omega^{v}\cdot p_{t}^{v}$$

where $p_{t}$ denotes the integrated prediction results from the multi-source teacher model, encompassing both the labeled data prediction outcomes $p_{t}\left(L_{t}\right)$ and the unlabeled data prediction outcomes $p_{t}\left(U_{t}\right)$.

### 3.4. Multi-Source Data Distillation Loss

When learning in task *t*, the model $G_{t}$ typically serves as the student model, while the model from task t−1, denoted as $G_{t-1}$. The distillation loss $L_{KL}$ between the output prediction probabilities of the teacher and student models is calculated using the KL divergence [24,25]. Introducing a temperature parameter T, the temperature-scaled probability distributions $P_{T}$ are shown in Equation (6):

(6) $$P_{T}=\frac{\exp\left(\frac{\log p_{t-1}^{1:c_{t-1}}}{T}\right)}{\sum_{c_{t-1}}\exp\left(\frac{\log p_{t-1}^{1:c_{t-1}}}{T}\right)}$$

Similarly, the temperature-scaled probabilities $P_{S}$ for the student model’s predictions are shown in Equation (7):

(7) $$P_{S}=\frac{\exp\left(\frac{\log p_{t}^{1:c_{t}}}{T}\right)}{\sum_{c_{t}}\exp\left(\frac{\log p_{t}^{1:c_{t}}}{T}\right)}$$

where $c_{t-1}$ represents the number of learned categories after task t−1. $p_{t-1}^{1:c_{t-1}}$ denotes the prediction probabilities output by the multi-source teacher models for the task t−1, and $p_{t}^{1:c_{t}}$ signifies the prediction probabilities output by the student model for the task *t*, *T* is the temperature parameter that controls the smoothness of the probability distribution.

Multi-source data distillation loss $L_{KL}$ are shown in Equation (8):

(8) $$L_{KL}=\sum P_{T}\log\left(\frac{P_{T}}{P_{S}}\right)$$

### 3.5. Total Loss Function

The total loss function $L_{t}$ for task *t* is composed of the cross-entropy loss function $L_{c}$ and the multi-source data distillation loss $L_{KL}$. The calculation formula for the loss function $L_{c}$ is shown in Equation (9):

(9) $$L_{c}=-\sum_{i=1}^{c_{t}}y_{t}^{i}\log\left(p_{t}\left(L_{t}\right)\right)$$

where $c_{t}$ represents the number of classes for the samples in task *t*, and $p_{t}\left(L_{t}\right)$ denotes the model’s prediction results after the fusion of labeled sample feature representations in task *t*.

The calculation formula for the total loss function $L_{t}$ is shown in Equation (10):

(10) $$L_{t}=L_{c}+\lambda L_{KL}$$

where λ is the weight of the multi-source data distillation loss function.

### 3.6. Semi-Supervised Enhanced Label Propagation Method

This paper employs a semi-supervised graph classification method based on enhanced label propagation for multiple data sources [26]. By integrating features from multiple data sources and utilizing a logistic regression classifier [27] to improve the label propagation algorithm, this method makes full use of the limited labeled data and a large amount of unlabeled data to enhance the accuracy of sucker-rod pumping well operating condition recognition. Specifically, the feature vectors $H^{\prime }\left(D\right)$ from different data sources are first fused using mean fusion. The feature matrix $X_{t}$ for task *t* after fusion is calculated as shown in Equation (11):

(11) $$X_{t}=\sum_{v=1}^{V}H^{l+1}\left(D_{t}^{v}\right)$$

where $X_{t}\in R^{K\times c_{t}}$ includes the global feature matrix $X_{L}$ for labeled data and the global feature matrix $X_{U}$ for unlabeled data, with $X_{t}=\left[X_{L},X_{U}\right]$. The semi-supervised graph classification method based on enhanced label propagation for multiple data sources is represented as shown in Equation (12):

(12) $$G_{t}=\left(A_{t},X_{t}\right)$$

where $A_{t}\in R^{K\times K}$ is the adjacency matrix constructed using the fused features, which includes the adjacency matrix $A_{L}$ built from labeled data and the adjacency matrix $A_{U}$ built from unlabeled data.

The adjacency matrix for $A_{kc}$ is constructed as Equation (13):

(13) $$A_{kc}=\left\{\begin{array}{cc} 1, & \mathrm{if}\;y_{k}=c\\ 0, & \mathrm{if}\;y_{k}\neq c \end{array}\right.$$

where $A_{kc}$ represents the probability that the *k*-th sample belongs to the *c*-th class, $y_{k}$ represents the class of *k*-th sample. The adjacency matrix for labeled data is $A_{L}=\sum_{k=1}^{K}\sum_{c=1}^{C}A_{kc}$.

The adjacency matrix for unlabeled data $A_{U}$ is constructed as Equation (14):

(14) $$A_{U}={\tilde{D}}_{t}^{-1/2}{\tilde{A}}_{t}{\tilde{D}}_{t}^{-1/2}$$

where ${\tilde{A}}_{t}=A_{t}+I_{K}$, with $I_{K}$ being the identity matrix of size K×K. ${\tilde{D}}_{t}$ is a diagonal matrix with elements ${\left({\tilde{D}}_{t}\right)}_{cc}=\sum_{k=1}^{K}{\left({\tilde{A}}_{t}\right)}_{ck}$.

The label propagation process is defined as Equation (15):

(15) $$H_{t}^{l+1}=\gamma \left(A_{L}+A_{U}\right)H_{t}^{l}+\left(1-\gamma \right)y_{t}$$

where γ is the propagation coefficient, which controls the extent of label propagation. $A_{L}$ is the adjacency matrix constructed from labeled data. $A_{U}$ is the adjacency matrix constructed from unlabeled data. $y_{t}$ is the label matrix of the labeled data. $H_{t}^{l+1}$ represents the label embedding vector after propagation.

This paper employs the logistic regression classifier to generate an initial prediction probability distribution for unlabeled data, providing a more accurate initial label vector for label propagation. After completing the label propagation process, multiple logistic regression classifiers $P_{LR}$ are used to classify the label embedding vectors, and the average of these classifiers’ results is calculated to obtain the final classification labels. The final labels $y_{t}$ for task *t* are calculated as shown in Equation (16):

(16) $$y_{t}=P_{LR}\left(H_{t}^{l+1}\right)$$

where $H_{t}^{l+1}$ is the label vector for task *t* after label propagation.

## 4. Experiment

### 4.1. Dataset

The experimental data comes from a high-pressure, low-permeability, and light oil reservoir block in a Chinese oilfield. The dataset from this area was used to construct a sucker-rod pumping well operating condition recognition dataset under the class-incremental learning scenario. The dataset was strictly selected according to the operation records of the oil wells, covering measured dynamometer cards (a binary image composed of displacement and load on the polish rod) and measured electric power cards (a binary image composed of polish rod displacement and effective electric power of the motor), reflecting the actual data points from the production site. The dataset accumulated condition samples from 60 pumping units over 3 years, totaling 1650 samples, covering 11 typical conditions, with each condition including 150 samples.

**Figure 3:**
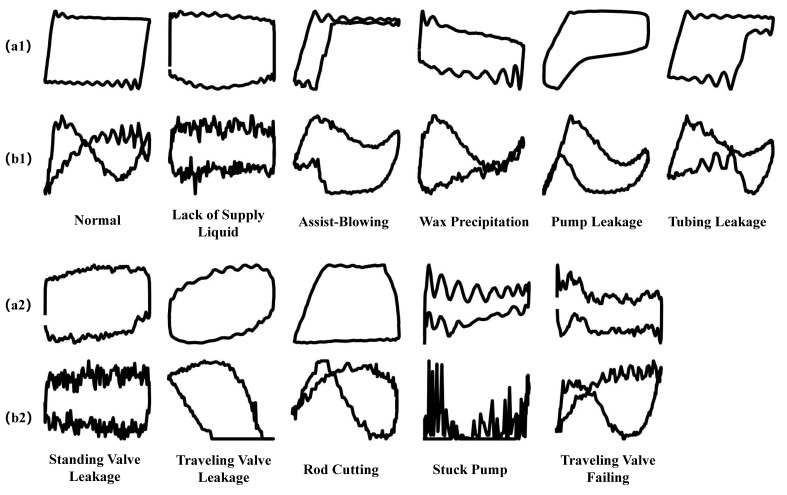
Dataset examples. (**a1**,**a2**) Measured ground dynamometer cards, (**b1**,**b2**) Measured electrical power cards.

The initial incremental learning task includes 2 categories, and each new task subsequently adds 1 or 2 categories. This design simulates the scenario where new operating conditions gradually appear in oilfield operations. Specifically, the 11 typical conditions in the dataset include: Normal, Lack of Supply Liquid, Assist-Blowing, Wax Precipitation, Pump Leakage, Tubing Leakage, Standing Valve Leakage, Traveling Valve Leakage, Rod Cutting, Stuck Pump, and Traveling Valve Failing. The dataset examples are shown in Figure 3.

The experimental environment for this paper is configured as follows: Python 3.8.10, PyTorch 1.11.0, CUDA 11.4, and a 24 GB memory NVIDIA RTX3090 GPU. The experimental design follows the actual operating conditions of oilfield pumping wells, with the initial conditions set to the commonly encountered “Normal” and “Lack of Supply Liquid” categories in oilfield production. To systematically evaluate the model’s performance under different labeled data ratios, this paper sets semi-supervised learning ratios at 10%, 30%, 50%, 70%, and 100%. The parameter *T* in Equation (6) is set to 2, λ in Equation (8) is set to 0.6, and all experimental results are based on the average of 10 runs to ensure accuracy and reliability.

### 4.2. Experimental Results

To evaluate the performance of our proposed method, we conducted a comparative analysis with the teacher model based on measured dynamometer cards (w/MDC-tech), the teacher model based on measured electrical power cards (w/MEPC-tech), and the method without the implementation of the multi-source data distillation (w/o MDD). Furthermore, this paper has reproduced two state-of-the-art class-incremental learning methods [28,29] and applied them to multi-source semi-supervised classification tasks. The experimental results are shown in Table 1.

From Table 1, it is evident that compared to methods without distillation learning and single-source teacher models, our proposed method achieves superior performance across various ratios of labeled data. The experimental results demonstrate that the fusion strategy not only enhances the model’s adaptability to multi-source data but also effectively captures complementary information between teacher models. Moreover, distillation learning effectively mitigates the model’s tendency to forget previously learned tasks. When compared with two class-incremental learning methods, the proposed method shows robustness in most scenarios with different ratios of labeled data and varying numbers of classes. In situations where labeled data is scarce (semi-supervised ratio of 10% with 11 classes), the accuracy of our method reaches 96.69%, which is a significant improvement over other methods. Under limited labeled data availability, our method efficiently capitalizes on the scarce labeled data while simultaneously harnessing the semi-supervised learning to make optimal use of unlabeled data, thereby boosting identification accuracy. As the proportion of labeled data increases, there is a corresponding improvement in the performance of all methods.

### 4.3. Ablation Study

#### 4.3.1. Analysis of Multi-Source Teacher Model Fusion Effects

To evaluate the effect of the multi-source teacher fusion method on the performance of class-incremental sucker-rod pumping well operating condition recognition, we designed and carried out comparative experiments under a semi-supervised ratio of 10%. The experiments encompassed three distinct configurations: w/MDC-tech, w/MEPC-tech, and the proposed multi-source teacher fusion method. By comparing the performance of these three different experimental setups, we explore the effectiveness of the multi-source data fusion strategy in enhancing the model’s recognition accuracy and robustness. The results show that our proposed method’s performance outperforms that of any single-source teacher model, with an improvement in accuracy of at least 0.5%. The experimental results demonstrate that the multi-source teacher model integration method can synthesize information from two data sources, providing a more comprehensive set of condition features and thereby increasing the accuracy and robustness of the sucker-rod pumping well operating condition recognition task. The experimental results are shown in Table 1.

#### 4.3.2. Influence of Different Attention on Multi-Source Data Fusion

To explore the effectiveness of different attention mechanisms in multi-source data fusion, this experiment compared three methods: the method using the average prediction probability as the weight (APP), the method using the maximum prediction probability as the weight (MPP), and the squeeze-and-excitation attention mechanism employed in this paper. The experimental results are shown in Table 2.

From Table 2, it can be observed that the Squeeze-and-Excitation attention mechanism used in this paper outperforms the other two methods in terms of accuracy. The Squeeze-and-Excitation attention mechanism is capable of dynamically adjusting the weights of different data sources, thereby more effectively capturing the complementary information between the data. In contrast, the direct weighted average method and the method using the maximum prediction probability as the weight may not fully leverage the differences between data sources in some cases, leading to limitations in model performance. The attention mechanism demonstrates higher recognition accuracy and robustness in the fusion of multi-source data due to its ability to respond flexibly to changes in data characteristics.

#### 4.3.3. Influence of Enhanced Label Propagation Method

To validate the effectiveness of the enhanced label propagation method, we conducted a comparative experiment with the traditional label propagation method. The experimental results are shown in Table 3.

As shown in Table 3, the enhanced label propagation method outperforms the traditional label propagation method in terms of performance. The logistic regression classifier generates an initial prediction probability distribution for the unlabeled data, providing a more accurate initial label vector for label propagation. This improvement significantly enhances the accuracy of label propagation, thereby achieving superior classification performance.

#### 4.3.4. Sensitivity Analysis of Multi-Source Data Distillation Fusion Loss

**Figure 4:**
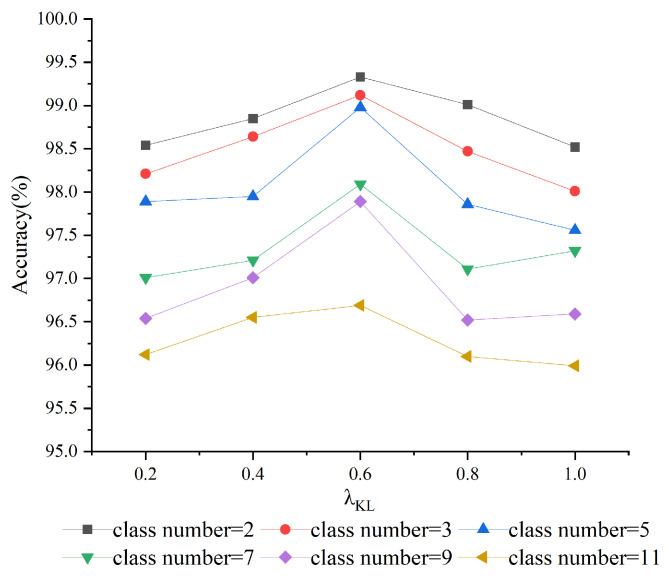
Performance of Different Distillation Fusion Loss Coefficients.

To explore the effect of the multi-source data distillation fusion loss coefficient and temperature coefficient T on model performance, we conducted experiments under a semi-supervised ratio of 10%. By adjusting the weight of the KL divergence within the total loss function, we analyzed the model’s performance on class-incremental learning tasks, with a specific focus on the impact of different coefficient settings on the retention of knowledge from previous tasks. Figure 4 presents the model’s recognition accuracy across various coefficient settings. The findings reveal that the model’s accuracy peaks as the weight increases; the model achieved its highest recognition accuracy when the weight was set at 0.6. These results suggest that there is an optimal weight that balances the model’s retention of old task knowledge with its ability to learn new tasks effectively, thus optimizing performance in class-incremental learning scenarios.

**Figure 5:**
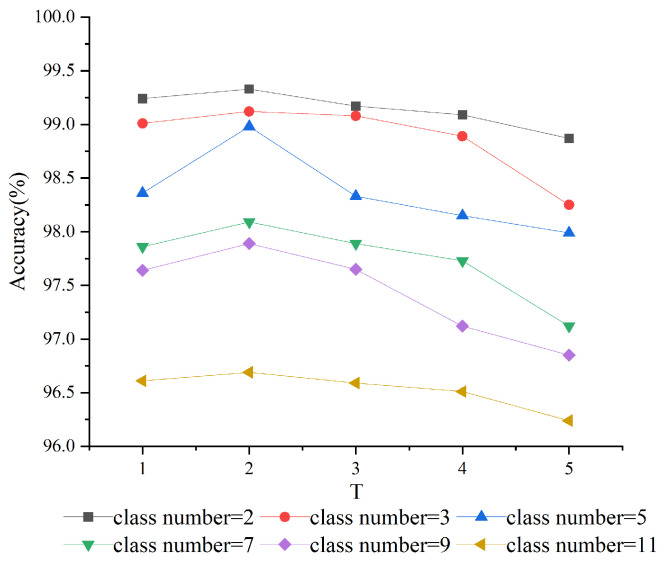
Performance of Different Distillation Fusion Temperature.

The temperature coefficient *T* controls the softening of output probabilities, which significantly impacts the effectiveness of knowledge transfer. To determine the optimal setting for our model, we tested various values of *T*. Figure 5 presents the model’s recognition accuracy across different temperature coefficient settings. Our results indicate that setting *T* to 2 provides the best balance between the smoothness of the output and the preservation of critical features from the multi-source data. This optimal value ensures that the model maintains high accuracy while effectively transferring knowledge across different tasks.

## 5. Conclusions

This paper proposes a semi-supervised class-incremental sucker-rod pumping well operating condition recognition method based on multi-source data distillation. First, we employ graph neural network to construct multi-source teacher models for measured data. Then, dynamically integrate prediction probabilities from different data sources using the squeeze-and-excitation attention mechanism, enhancing the model’s adaptability to multi-source data. Subsequently, we introduce multi-source data distillation loss to effectively alleviate catastrophic forgetting of prior knowledge during class-incremental learning. Finally, we improve the label propagation algorithm with a logistic regression classifier, deeply exploring the potential relationship between labeled. The experimental results show that the proposed method achieves superior performance on the class-incremental sucker-rod pumping well operating condition recognition dataset, significantly improving the accuracy and robustness.

The research achievements of this paper have made certain progress in incremental oil well operating condition recognition, but the following directions can be further explored: Conduct in-depth research on existing multi-source data fusion strategies, explore more efficient algorithm optimizations, and enhance the model’s processing and adaptability to multi-source data.

## Figures and Tables

**Table 1:** Performance Comparison of Sucker-rod Pumping Well Operating Condition Recognition.

Label Proportion	Methods	2	3	5	7	9	11
10%	w/o MDD	97.66	97.57	97.41	97.01	96.16	95.47
	w/MDC-tech	98.66	98.51	97.98	97.49	96.74	96.03
	w/MEPC-tech	98.34	97.64	97.81	97.43	96.17	95.71
	MDPCR	98.80	98.69	98.62	97.91	98.48	95.81
	PSM	98.86	98.74	98.66	97.73	96.85	96.19
	Ours	99.33	99.12	98.98	98.09	97.89	96.69
30%	w/o MDD	98.34	97.85	97.67	97.29	96.59	96.22
	w/MDC-tech	98.89	98.66	97.94	98.02	97.59	96.99
	w/MEPC-tech	98.72	97.88	97.83	97.65	96.66	96.38
	MDPCR	99.00	98.79	98.41	98.45	98.16	97.63
	PSM	99.15	98.91	98.87	98.64	98.24	97.55
	Ours	99.47	99.30	99.15	98.98	98.35	98.29
50%	w/o MDD	98.40	98.16	97.98	97.99	97.72	96.58
	w/MDC-tech	99.11	98.72	98.02	98.10	97.88	97.25
	w/MEPC-tech	99.07	98.68	97.98	98.05	97.79	96.89
	MDPCR	99.22	99.08	98.83	98.78	98.59	98.40
	PSM	99.30	99.17	98.88	98.89	98.64	98.37
	Ours	99.51	99.34	99.28	99.17	98.85	98.72
70%	w/o MDD	98.49	98.24	98.17	98.09	97.88	97.11
	w/MDC-tech	99.25	98.82	98.22	98.14	98.05	97.36
	w/MEPC-tech	99.23	98.76	98.18	98.11	97.99	97.14
	MDPCR	99.36	99.28	98.98	98.83	98.65	98.55
	PSM	99.45	99.32	99.11	99.13	98.71	98.77
	Ours	99.61	99.45	99.39	99.19	98.98	98.84
100%	w/o MDD	98.67	98.33	98.21	98.17	97.93	97.26
	w/MDC-tech	99.45	99.21	99.15	98.52	98.84	97.70
	w/MEPC-tech	99.35	99.17	99.03	98.49	98.75	97.33
	MDPCR	99.49	99.37	99.15	98.92	99.04	98.61
	PSM	99.56	99.44	99.26	99.17	98.93	98.83
	Ours	99.78	99.57	99.41	99.33	99.03	98.99

**Table 2:** Influence of Different Attention Mechanisms on Multi-Source Data Fusion.

Methods	2	3	5	7	9	11
w/APP	98.33	98.13	97.92	97.22	96.67	95.21
w/MPP	98.66	98.42	98.19	97.79	97.01	95.83
Ours	99.33	99.12	98.98	98.09	97.89	96.69

**Table 3:** Influence of Enhanced Label Propagation Method.

Methods	2	3	5	7	9	11
Label Propagation	97.18	97.03	96.66	95.62	95.89	93.95
Ours	99.33	99.12	98.98	98.09	97.89	96.69

## Data Availability

The data supporting this study’s findings are available from the corresponding author, B.Z., upon reasonable request.

## Appendix A

The Table A1 shows the key symbols and variables used in this manuscript.

**Table A1:** Notation description.

Notation	Description
*D*	Multi-source class-incremental dataset
*V*	The number of information sources
*T*	The number of class-incremental learning tasks
$L_{t}^{v}$	Labeled data set of the $v^{th}$ data source in task *t*
$U_{t}^{v}$	Unlabeled data set of the $v^{th}$ data source in task *t*
*k*	The number of labeled data
*m*	The number of unlabeled data
*A*	Adjacency matrix of the GCN
*H*	Feature matrix of the GCN
*W*	Learnable parameter matrix in the GCN
$H_{t}^{l+1}$	Label embedding vector after propagation
*y*	Corresponding label for the samples
$\hat{y}$	Predicted label vector for the samples
$p_{t-1}^{1:c_{t-1}}$	Prediction probability output by the teacher model
$p_{t}^{1:c_{t}}$	Prediction probability output by the student model
$P_{T}$	Temperature-scaled probability output by the teacher model
$P_{S}$	Temperature-scaled probability output by the student model
$L_{t}$	Total loss function
$L_{c}$	Cross-entropy loss function
$L_{KL}$	Kullback-Leibler divergence loss function
λ	Weight of the multi-source data distillation loss function
